# Impact of a Community-Based Lymphedema Management Program on Episodes of Adenolymphangitis (ADLA) and Lymphedema Progression - Odisha State, India

**DOI:** 10.1371/journal.pntd.0003140

**Published:** 2014-09-11

**Authors:** Katherine E. Mues, Michael Deming, David G. Kleinbaum, Philip J. Budge, Mitch Klein, Juan S. Leon, Aishya Prakash, Jonathan Rout, LeAnne M. Fox

**Affiliations:** 1 Department of Epidemiology, Rollins School of Public Health and Laney Graduate School, Emory University, Atlanta, Georgia, United States of America; 2 Parasitic Diseases Branch, Division of Parasitic Diseases and Malaria, Center for Global Health, Centers for Disease Control and Prevention, Atlanta, Georgia, United States of America; 3 Department of Epidemiology, Rollins School of Public Health, Emory University, Atlanta, Georgia, United States of America; 4 Church's Auxiliary for Social Action, Odisha, India; Institute of Medical Microbiology, Immunology and Parasitology, Germany

## Abstract

**Background:**

Lymphedema management programs have been shown to decrease episodes of adenolymphangitis (ADLA), but the impact on lymphedema progression and of program compliance have not been thoroughly explored. Our objectives were to determine the rate of ADLA episodes and lymphedema progression over time for patients enrolled in a community-based lymphedema management program. We explored the association between program compliance and ADLA episodes as well as lymphedema progression.

**Methodology/Principal Findings:**

A lymphedema management program was implemented in Odisha State, India from 2007–2010 by the non-governmental organization, Church's Auxiliary for Social Action, in consultation with the Centers for Disease Control and Prevention. A cohort of patients was followed over 24 months. The crude 30-day rate of ADLA episodes decreased from 0.35 episodes per person-month at baseline to 0.23 at 24 months. Over the study period, the percentage of patients who progressed to more severe lymphedema decreased (P-value  = 0.0004), while those whose lymphedema regressed increased over time (P-value<0.0001). Overall compliance to lymphedema management, lagged one time point, appeared to have little to no association with the frequency of ADLA episodes among those without entry lesions (RR = 0.87 (0.69, 1.10)) and was associated with an increased rate (RR = 1.44 (1.11, 1.86)) among those with entry lesions. Lagging compliance two time points, it was associated with a decrease in the rate of ADLA episodes among those with entry lesions (RR = 0.77 (95% CI: 0.59, 0.99)) and was somewhat associated among those without entry lesions (RR = 0.83 (95% CI: 0.64, 1.06)). Compliance to soap was associated with a decreased rate of ADLA episodes among those without inter-digital entry lesions.

**Conclusions/Significance:**

These results indicate that a community-based lymphedema management program is beneficial for lymphedema patients for both ADLA episodes and lymphedema. It is one of the first studies to demonstrate an association between program compliance and rate of ADLA episodes.

## Author Summary

Lymphatic filariasis (LF) is characterized by clinical manifestations of limb swelling, lymphedema, and elephantiasis. LF is the world's second-leading cause of disability, with up to 15 million people with limb lymphedema or elephantiasis. The Global Programme to Eliminate LF aims to eliminate the disease through two pillars: (1) interruption of transmission and (2) treatment of clinical disease among those already affected. The Church's Auxiliary for Social Action (CASA), an Indian NGO, began a lymphedema management program in Khurda District, Odisha State, India in 2007. We evaluated the impact of the program on two clinical LF outcomes: acute episodes of adenolymphangitis (ADLA) and chronic lymphedema progression. The monthly rate of ADLA episodes decreased significantly after enrollment for two years in the program. Additionally, program compliance was found to be associated with ADLA rate among some clinically distinct groups. Compliance to soap was associated with a decreased rate of ADLA episodes among those without inter-digital entry lesions. The percentage of patients who progressed to more severe lymphedema decreased significantly over time, while those whose lymphedema regressed increased significantly. These results indicate that a community-based lymphedema management program is beneficial for lymphedema patients for both ADLA episodes and lymphedema. It is one of the first studies to demonstrate an association between program compliance and ADLA episodes.

## Introduction

Lymphatic filariasis (LF), caused by parasitic nematode worms, is characterized by clinical manifestations of swelling, lymphedema, and elephantiasis of the limbs. These thread-like worms reside in the lymphatic vessels of humans, resulting in severe lymphatic damage and dysfunction. Persons with lymphatic dysfunction caused by LF may suffer from adenolymphangitis (ADLA) episodes characterized by swelling, inflammation of the limbs, fever, malaise and chills [1]. The frequency of ADLA episodes has been shown to increase with more advanced lymphedema and studies have concluded that ADLA episodes are a risk factor for lymphedema progression [2]–[6].

LF affects over 120 million people throughout the world with 1.3 billion at risk [7]. India accounts for over 40% of the global LF burden [8], with an estimated 40 million persons infected [1] and 7 million with chronic lymphedema. India has a national goal of LF elimination by 2015. The goals of the elimination program are to 1) interrupt transmission and 2) control morbidity among infected persons [9]. To interrupt transmission, the entire at-risk population must be treated with microfilariae-killing drugs through yearly mass drug administration (MDAs). To control morbidity, lymphedema management programs focus on basic limb hygiene to prevent ADLA episodes and stop further progression of lymphedema. The World Health Organization (WHO) has recently recommended that all LF endemic countries provide access to lymphedema management services [10].

Lymphedema management programs have been shown to decrease the frequency of ADLA episodes and lymphedema severity both in clinical-trial [11], [12] and field settings [13], [14]. Patients enrolled in a clinic-based lymphedema management program in Léogâne, Haiti demonstrated significant decreases in the incidence of ADLA episodes and reduction of leg volume over a 4-year period [13]. A study of a home-based lymphedema management program in Burkina Faso also demonstrated significant reductions in the percent of patients experiencing at least one ADLA episode over a month's time [14]. The studies completed to date vary in their methods of data collection, follow-up intervals, and study length. There are no studies on home-based lymphedema management programs that calculate an ADLA rate and explore the effects of lymphedema programs using longitudinal models. Most studies did not adjust for potential confounding or test for interaction. Finally, there are few studies exploring the association between compliance to lymphedema management techniques and clinical outcomes of LF.

Following implementation of a community-based lymphedema management program in India, the aim of this study was to examine the rate of ADLA episodes and progression of lymphedema over a two year period. It also aimed to determine predictors of compliance to the program and assess the effect of compliance on the rate of ADLA episodes and lymphedema progression.

## Methods

### Ethics Statement

This project was submitted for human subjects review to the Center for Global Health at the Centers for Disease Control and Prevention (CDC), Atlanta, Georgia, USA. It was approved by CDC and determined to be program evaluation. Permission for the survey was obtained from the Odisha State Department of Health and Family Welfare. Participants were asked to give their written informed consent prior to participation. For those unable to write, consent was documented by recording the person's fingerprint or marking the signature line with an ‘X’ and by countersignature of survey personnel. For participants under 18 years of age, verbal consent of a parent or guardian was also obtained. Consent procedures were approved by CDC and the Odisha State Department of Health and Family Welfare.

### Study Area

The lymphedema management program was implemented in Khurda district, Odisha State, India by the non-governmental organization, Church's Auxiliary for Social Action (CASA). Khurda district has a population of nearly 2.2 million and is highly endemic for LF caused by *Wuchereria bancrofti*. Based on 2001 estimates, there were 3.5 million – 6.3 million infected persons in Odisha state and 22,500–23,500 infected persons in Khurda district [15]–[18].

### Lymphedema Management Program

All patients enrolled in the program were trained in basic lymphedema management by physician-trained volunteers, including daily washing of limbs with soap and water, daily exercise and elevation of the affected limb, and daily use of footwear outside the home. Patients were trained in the importance of early treatment and prevention of secondary bacterial and fungal infections with topical and oral antimicrobial agents. If inter-digital fungal infections were present, patients were instructed to use an antifungal cream on a daily basis. Patients were supplied with soap and antifungal cream for the first 6 months of the program; thereafter they were instructed to purchase these supplies at local stores and pharmacies.

### Study Design

To evaluate CASA's community-based lymphedema management program, a cohort of individuals enrolled in the program was recruited. Of 189 villages in Bologarh block of Khurda district, 30 villages were eligible for inclusion in the study as they had not yet been enrolled in the lymphedema management program and were not located in the immediate vicinity of an already enrolled village. In the 30 selected villages, 533 persons with lymphedema were identified in June 2009. Of these patients, 456 persons were approached to be in the lymphedema program, as some patients had migrated out of their village during the 5–6 month period since the initial household census.

Enrollment eligibility included persons more than 14 years of age who reported lower leg swelling for at least 3 months. Of the 456 persons approached, 375 (82%) met the inclusion criteria and agreed to participate. Five patients were subsequently excluded from the analysis due to lack of lymphedema on examination (n = 2), failure to meet age criteria (n = 1), or mislabeling of survey forms (n = 2) for a total sample size of 370 (81%). There were no differences in the distribution of age, sex, and lymphedema stage among those who did not enroll compared to those who did enroll in the study (data not shown).

Patients were interviewed in Oriya (the local language) by trained interviewers at baseline (prior to enrollment) and at, 1, 2, 3, 6, 12, 18 and 24 months after enrollment in the program beginning in July 2009. We did not include a true control population in this study as it may be considered unethical to deny a person suffering from lymphedema access to a lymphedema management program as is recommended by the WHO for all LF endemic countries [10]. To evaluate the effectiveness of the program over time, we consider baseline, before patients were enrolled in the program, as the comparison group. Through a written questionnaire, interviewers collected information on patient demographics, frequency of compliance to lymphedema management techniques, ADLA history and treatment, access to supplies, MDA history, and perceived disability using the World Health Organization Disability Assessment Schedule II (WHO-DAS II). Interviewers also completed a clinical assessment on each person in order to determine lymphedema stage.

Data were independently dual-entered into an Epi Info 7 (Stone Mountain, 2008) database. Data cleaning and analysis were performed in SAS 9.3 (Cary, North Carolina, USA). A sample size of 375 was calculated to detect a 5% decrease in the frequency of ADLA episodes, with a 15% dropout rate, from baseline to 24 months post-enrollment.

### Definitions

#### 30-day rate of ADLA episodes

An ADLA episode was defined by patient self-report of two or more of the following symptoms: redness, pain, or swelling of the leg or foot, with or without the presence of fever or chills, [3], [19]. Patients were asked how many times they had an ADLA episode in the previous 30 days. The ADLA rate per subject was calculated as the number of ADLA episodes reported by each subject divided by 30. We assumed that each ADLA episode reported initially occurred during the 30-day period and that each person remained at risk for ADLA episodes during the entire 30-day retrospective period.

#### Lymphedema progression

Both the interviewer and a supervisor performed independent lymphedema staging and photographs were taken of the affected limb(s). The 7-stage classification system [20] was used to stage lymphedema. Any discrepancies between interviewer and supervisor staging were resolved by independent evaluation of limb photographs by two physicians with extensive LF experience (P. Budge and L. Fox). Patients whose lymphedema stage increased from one time point to another were determined to have progressed, while those whose lymphedema stage decreased from one time point to another were determined to have regressed. Lymphedema stages 1–3 were categorized as early; stages 4–7 were categorized as advanced.

#### Compliance to lymphedema management techniques

Compliance with specific lymphedema management techniques was measured by self-report. Patients were asked how frequently they performed each of five management techniques: washing affected leg with soap and water, treating inter-digital entry lesions with antifungal cream, elevating the limb, exercising the limb, and wearing footwear outside. CASA's program did not provide oral antimicrobials for ADLA episodes; therefore, compliance with oral antibiotics was not measured. A person was considered to be compliant to a lymphedema management technique if he/she reported performing that technique at least once per day.

An overall weighted compliance score for soap, cream, elevation, exercise, and wearing footwear outside was created. If patients reported performing a technique daily or more than once per day, they received a score of 2. If they reported performing a technique once per week or more than once per week, they received a score of 1, and if they reported performing a technique less frequently than once per week, they received a score of 0. Since cream was only indicated for patients with inter-digital entry lesions present, those without entry lesions had a potential maximum score of 8. To make the score of these patients comparable to those with entry lesions, the scores of patients without entry lesions were multiplied by a factor of 1.25. The scores for each technique were then summed for a maximum of 10. The summary score was then divided into two groups: compliant  = 7–10 and non-compliant  = 0–6.

### Statistical Analysis

To determine how the presence of the lymphedema program influenced the number of ADLA episodes experienced per person per month, Poisson models for correlated data (up to 8 observations per subject) were built with SAS's PROC GLIMMIX using an auto-regressive (1) correlation structure with a random intercept. The model contained dummy variables for time, variables for presence of inter-digital entry lesions, lymphedema status at baseline (advanced vs. early), and number of times a patient had participated in a MDA as this has been shown to be associated with the frequency of ADLAs episodes [21]. Interaction terms involving time with presence of entry lesions and lymphedema status at baseline were also included in the model. After assessment of interaction, confounding and precision [22], an adjusted rate ratio was calculated comparing the 30-day rate of ADLA episodes at each time point to baseline.

To evaluate the association between compliance to lymphedema management techniques and the 30-day rate of ADLA episodes, a mixed effects Poisson model for correlated data was executed. Overall compliance was evaluated through the compliance score while compliance to each technique was defined dichotomously (compliant vs. non-compliant); both were lagged one time point. Each model used an auto-regressive (1) correlation structure and included a random intercept. The models controlled for confounding by access to water, soap, antifungal cream, and a hospital, as well as number of times a patient had participated in a MDA. Models of compliance to individual lymphedema management techniques also controlled for compliance to all other techniques at the lagged time point. Interaction by baseline lymphedema stage (advanced vs. early) and presence of inter-digital entry lesions at the lagged time point was assessed testing product terms with compliance. If no significant interaction was found, main effects of baseline lymphedema status and/or presence of entry lesions were included in the model.

To explore the association between compliance to lymphedema management techniques and lymphedema progression, a fixed effects logistic model for correlated data with an auto-regressive (1) correlation structure was used. We explored both overall compliance and compliance to each technique lagged one time point, adjusting for the number of times a patient had participated in a MDA. Models of compliance to individual lymphedema management techniques also controlled for compliance to all other techniques at the lagged time point unless otherwise noted. Interaction by baseline lymphedema stage (advanced vs. early) and presence of inter-digital entry lesions at the previous time point was assessed testing product terms with compliance. If no significant interaction was found, main effects of baseline lymphedema status and/or presence of entry lesions at the previous time point were also included in the model.

Confounding was evaluated using both *a priori* causal pathways and data-driven methods. Only combinations of confounders that fell within 10% of a gold standard model with all potential confounders were considered for the final model.

To explore predictors (all of which were considered exposure variables) of compliance to lymphedema management techniques, correlated mixed effects logistic models were used. All models used an auto-regressive (1) correlation structure with a random intercept. Potential predictors of compliance that were statistically significant at alpha = 0.05 in bivariate models were included in a multivariable model.

## Results

### Baseline Demographic Characteristics

Table 1 displays the baseline demographic characteristics of the 370 lymphedema patients enrolled at baseline. The mean age was 57 years and the majority (59%) of the cohort was female. At baseline, about 27% of patients had inter-digital entry lesions, 14% had advanced stage lymphedema (stages 4–6), and patients reported having lymphedema symptoms for an average of 25 years.

**Table 1:** Baseline characteristics of patients enrolled in a lymphedema management program, Khurda District, Odisha State, India, July 2009.

	N (mean)	% (SD)
**Age**	57	13.93
**Female Gender**	218	58.92
**Education**		
None at all	143	38.65
Completed at least primary school	226	61.08
**Occupational Status**		
** **Paid work	120	32.43
** **Non-paid work or unemployed	235	63.51
**Caste**		
General Caste	147	39.73
Other Backward Caste	175	47.30
Scheduled Caste	28	7.57
Scheduled Tribe	20	5.41
**Presence of Chronic Health Condition other than Lymphedema**	162	43.78
**Comorbidities**		
** **High blood pressure	62	16.76
Diabetes	12	3.24
Cancer	2	0.54
Heart problems	8	2.16
Stomach problems	66	17.84
**Inter-digital Entry Lesions Present**	100	27.03
**Advanced Lymphedema Stage (4–6)**	53	14.32
**Years with Lymphedema**	25	16.04

(N = 370).

Fifty-four (14.6%) patients were lost to follow-up during the 24 month study period; these patients did not contribute data at the 24 month time point. Over the course of the study, reasons for non-participation at any particular assessment were: absence from the village at the time of the assessment (70%), refusal (7%), illness (6%), or death (17%). In total, the study encompassed over 222 person-years of observation time (baseline to time of last follow-up). Two hundred thirty five patients (63.5%) contributed data at all 8 time points, 76 (20.5%) contributed data at 7 time points, and 29 (7.8%) contributed data at 6 time points. Only about 8% of patients contributed data at 5 time points or less. Censoring (i.e. contributing data at less than 8 time points) was not significantly associated with the rate of ADLA episodes (RR = 0.89 (0.52, 1.53)) nor with lymphedema stage progress (OR = 0.83 (0.61, 1.14)). Therefore, we assume that censoring was independent of the outcomes of interest.

During the two year study period, MDAs occurred in Odisha in June 2010 and March 2011 (Odisha State Ministry of Health and Family Welfare).

### 30-Day Rate of ADLA Episodes

Over the follow-up period, patients reported a total of 687 ADLA episodes. The sum of ADLA episodes for each person over the follow-up period ranged from 0–24, with a median of 1, a mean of 1.86, and an inter-quartile range of 1–3. Looking at the distribution of the sum of ADLA episodes over the study period, 44% of patients experienced 0 episodes, 17% experienced 1 episode, 21% experienced 2–3 episodes, and 19% experienced 4 or more episodes. The total number of reported episodes reported ranged from 129 at baseline to 45 at 6 months and was 72 at 24 months (Table 2). The mean duration of ADLA episodes reported was 3.5 days.

**Table 2:** 30-day rate of ADLA over time among lymphedema management participants.

Time (months)	N	# episodes	ADLA Rate per person-month	Rate Ratio (95% CI)*	Adjusted Rate Ratio (95% CI)**
0	370	129	0.35	1.00	1.00
1	351	92	0.26	0.68 (0.54, 0.85)	0.68 (0.54, 0.86)
2	349	101	0.29	0.74 (0.59, 0.94)	0.73 (0.58, 0.93)
3	339	88	0.26	0.64 (0.50, 0.81)	0.63 (0.49, 0.80)
6	324	45	0.14	0.34 (0.25, 0.46)	0.32 (0.24, 0.44)
12	321	74	0.23	0.58 (0.45, 0.75)	0.57 (0.44, 0.74)
18	332	86	0.26	0.66 (0.51, 0.84)	0.65 (0.51, 0.84)
24	316	72	0.23	0.57 (0.44, 0.74)	0.57 (0.43, 0.74)

*Rate ratios were calculated using mixed Poisson models accounting for random effect of subject, using an auto-regressive (1) correlation structure. The models used data at the individual level.

**Adjusted for patient report of number of MDAs in which they had participated.

**Figure 1:**
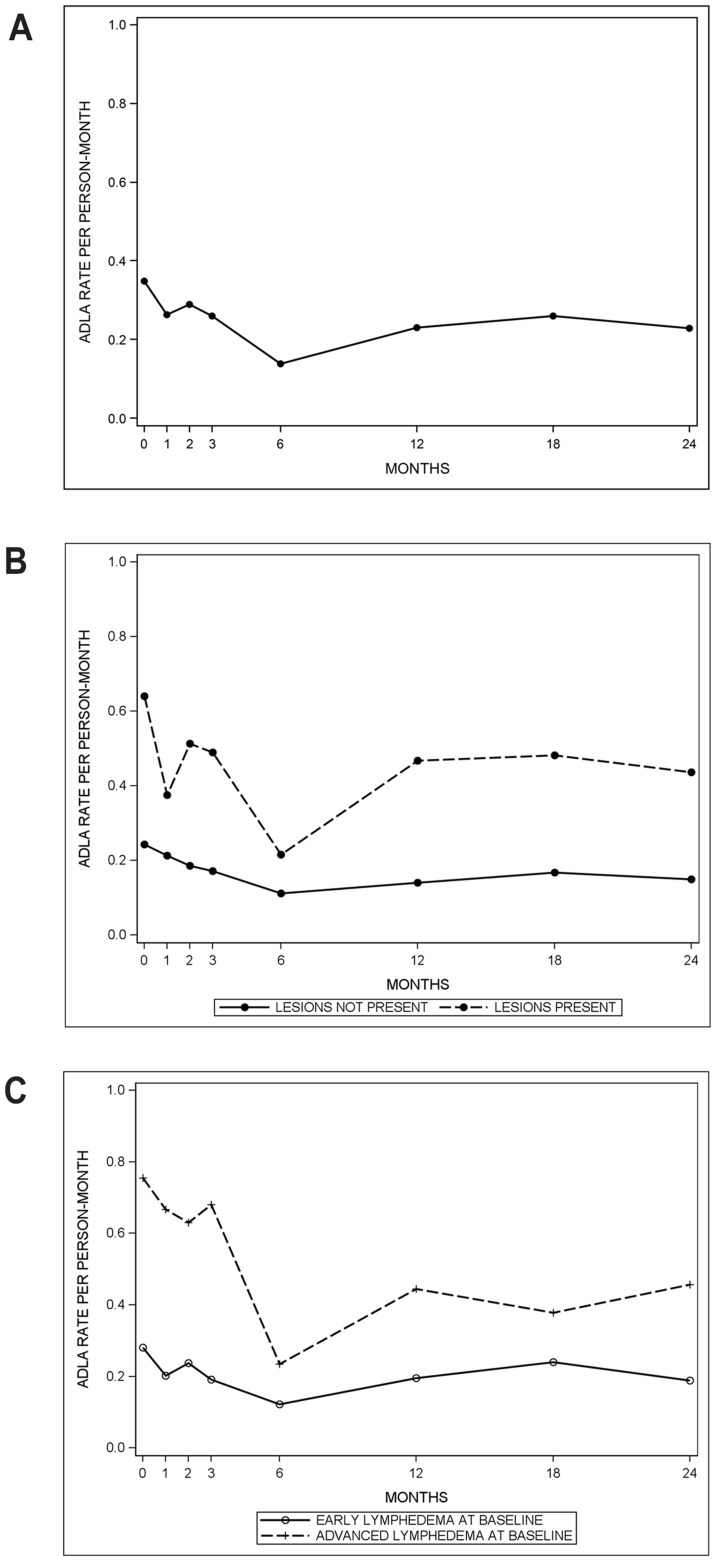
30-day rate of ADLA episodes, Khurda District, Odisha State, India. July 2009–July 2011. A. Overall 30-day rate of ADLA episodes. B. 30-day rate of ADLA episodes stratified by presence of inter-digital entry lesions. C. 30-day rate of ADLA episodes stratified by baseline lymphedema status: early lymphedema (stages 1–3), advanced lymphedema (stages 4–7).

The rate of ADLA episodes (Figure 1a) decreased from 0.35 episodes per person-month at baseline to 0.14 at 6 months and plateaued to 0.23 at 24 months, representing a 35% decrease. Those who had entry lesions had a higher rate of ADLA compared to those who did not (data not shown) (RR = 1.88 (95% CI: 1.54, 2.29)), and those with advanced lymphedema at baseline had a higher rate of ADLA compared to those with early lymphedema at baseline (RR = 2.66 (95% CI: 1.89, 3.75)).

Figure 1b displays the 30-day rate of ADLA episodes by the presence of inter-digital entry lesions. Those with lesions have a higher rate of ADLA episodes at each time point and also appear to have a greater difference when comparing the 24-month rate to the baseline rate. Figure 1c displays the 30-day rate of ADLA episodes by lymphedema status at baseline. Those with advanced lymphedema at baseline have a higher rate of ADLA episodes at each time point.

Table 2 displays a Poisson model exploring the effect of time on the individual ADLA rate controlling for number of MDAs and taking into account the correlated nature of the data. The rate ratios (RR) compare the rate at each time point to baseline. All of the RRs were less than 1 and statistically significant. The RR with greatest magnitude was seen at 6 months. Comparing 24 months to baseline, the rate of ADLA episodes was 0.57 that of the rate at baseline. In the Poisson model, we found significant interaction by baseline lymphedema status (advanced vs. early) and the presence of inter-digital entry lesions at the current time point. The stratified rate ratios (RR) of the final Poisson model controlling for the number of times a patient participated in a MDA are displayed in Table 3. Those with advanced lymphedema and entry lesions experienced a 43% reduction in ADLA episodes from baseline to 24 months (RR = 0.57 (95% CI: 0.35, 0.93)). Among those with advanced lymphedema and no entry lesions, the 30-day rate of ADLA episodes decreased by 62% (RR = 0.38 (95% CI: 0.17, 0.82)). Among those with early lymphedema, those with entry lesions had a 21% reduction in ADLA episodes (RR = 0.79 (95% CI: 0.46, 0.95)), while those without entry lesions experienced a 48% reduction (RR = 0.52 (95% CI: 0.35, 0.76)).

**Table 3:** 30-day rate of ADLA over time among lymphedema study participants by presence of inter-digital entry lesions at current time point and lymphedema status at baseline, Khurda District, Odisha State, India, July 2009–July 2011.

Time	N†	# episodes	Observed ADLA Rate per person-month	Adjusted Rate Ratio (95% CI)*	N†	# episodes	Observed ADLA Rate per person-month	Adjusted Rate Ratio (95% CI)*
	**Advanced Lymphedema at Baseline: Entry Lesions Present**				**Advanced Lymphedema at Baseline: No Entry Lesions**			
0	43	38	0.88	1.00	9	2	0.22	1.00
1	37	27	0.73	0.77 (0.50, 1.17)	8	3	0.38	1.12 (0.58, 2.16)
2	37	25	0.68	0.70 (0.45, 1.10)	8	4	0.50	0.54 (0.27, 1.06)
3	39	29	0.74	0.78 (0.51, 1.19)	8	3	0.38	0.77 (0.38, 1.55)
6	36	5	0.14	0.26 (0.14, 0.49)	11	6	0.55	0.27 (0.11, 0.67)
12	37	19	0.51	0.54 (0.33, 0.88)	8	1	0.13	0.31 (0.15, 0.66)
18	41	17	0.41	0.45 (0.27, 0.75)	4	0	0.00	0.22 (0.10, 0.49)
24	38	20	0.53	0.57 (0.35, 0.93)	7	1	0.14	0.38 (0.17, 0.82)
	**Early Lymphedema at Baseline: Entry Lesions Present**				**Early Lymphedema at Baseline: No Entry Lesions**			
0	57	26	0.46	1.00	258	63	0.24	1.00
1	75	15	0.20	0.49 (0.29, 0.81)	226	47	0.21	0.71 (0.51, 1.00)
2	74	32	0.43	0.86 (0.55, 1.36)	228	40	0.18	0.66 (0.46, 0.95)
3	55	17	0.31	0.56 (0.33, 0.94)	237	39	0.16	0.56 (0.39, 0.80)
6	43	12	0.28	0.35 (0.17, 0.69)	231	21	0.09	0.35 (0.23, 0.54)
12	55	24	0.44	0.83 (0.51, 1.35)	212	30	0.14	0.48 (0.32, 0.72)
18	44	24	0.55	1.16 (0.70, 1.92)	234	40	0.17	0.58 (0.41, 0.84)
24	49	18	0.37	0.79 (0.46, 0.95)	220	33	0.15	0.52 (0.35, 0.76))

† The sample size at each time point may not add up to 370 because of missing data and/or lost to follow-up.

*Adjusted results of Poisson regression for correlated data with significant interaction by entry lesions and lymphedema status. Controlling for number of MDAs in which patients reported participating.

### Lymphedema Progression

The overall distribution of lymphedema stage, as classified in Table 4, changed significantly from baseline to 24 months (p-value  = 0.0027). The percentage of patients with stage 3 or 4 lymphedema decreased over the study period (p-value  = 0.0006) while the percentage of patients with stage 1 or 2 lymphedema increased (p-value  = 0.0064) over the study period (Table 4). The small change in the percentage of patients with stage 5 or 6 lymphedema was not statistically significant. The percentage of patients who progressed to more severe lymphedema since the previous time point decreased (Table 4) (p-value  = 0.0004), while the percentage of those whose lymphedema regressed increased over time (p-value<0.0001). Patients with advanced lymphedema at baseline were less likely to progress to a more severe lymphedema stage (data not shown); OR = 0.15 (95% CI: 0.07, 0.36). Patients with entry lesions at each time point were also less likely to progress, OR = 0.55 (95% CI: 0.38, 0.79), yet more likely to have advanced lymphedema, OR = 1.17 (95% CI: 1.08, 1.26).

**Table 4:** Lymphedema progression over the study period, Khurda District, Odisha State, India, July 2009–July 2011.

	Baseline	1 Month	2 Month	3 Month	6 Month	12 Month	18 Month	24 Month	P-value*
	N (%)	N (%)	N (%)	N (%)	N (%)	N (%)	N (%)	N (%)	
Lymphedema Stage									
1–2	184 (48.73)	166 (47.29)	183 (52.44)	181 (53.39)	175 (54.01)	179 (55.76)	173 (52.11)	190 (60.13)	0.0064
3–4	139 (37.57)	146 (41.60)	126 (36.10)	115 (33.92)	105 (32.41)	97 (30.22)	115 (34.64)	80 (25.32)	0.0006
5–6	47 (12.70)	39 (11.11)	40 (11.46)	43 (12.68)	44 (13.58)	45 (14.02)	44 (13.25)	46 (14.56)	0.4795
Lymphedema Progression Since Previous Time Point									
Progression	NA	46 (13.11)	27 (7.74)	16 (4.72)	18 (5.56)	28 (8.72)	42 (12.65)	16 (5.06)	0.0004
No change	NA	278 (79.20)	284 (81.38)	301 (88.79)	283 (87.35)	264 (82.24)	266 (80.12)	240 (75.95)	0.3139
Regression	NA	27 (7.69)	38 (10.89)	22 (6.49)	23 (7.10)	24 (7.23)	24 (7.23)	60 (18.99)	<0.0001
Lymphedema Progression Since Baseline									
Progression	NA	46 (13.11)	44 (12.61)	42 (12.39)	36 (11.11)	43 (13.40)	55 (16.57)	29 (9.18)	0.1088
No change	NA	278 (79.20)	268 (76.79)	257 (75.81)	245 (75.62)	227 (70.72)	232 (69.88)	218 (68.99)	0.0026
Regression	NA	27 (7.69)	37 (10.60)	40 (11.80)	43 (13.27)	51 (15.89)	45 (13.55)	69 (21.84)	<0.0001

*Denotes the p-value for difference between baseline and 24 months.

### Compliance to Lymphedema Management Techniques

Compliance to all lymphedema management techniques measured through the compliance score and dichotomized into two categories increased from 3% at baseline to 74% at 6 months and plateaued to 63% at 24 months (p-value 24 months vs. baseline <0.0001) (Table 5). Compliance to soap use increased rapidly and remained high throughout the study (92%) (data not shown). Among those with inter-digital entry lesions, compliance to antifungal cream increased from 8% at baseline to 55% at 24 months (p-value <0.0001). Compliance to elevation, exercise, and wearing footwear outside the home reached moderate levels at 24 months: 88%, 62%, and 51% respectively.

**Table 5:** Distribution of program compliance and potential predictors of compliance to lymphedema management techniques over time among patients enrolled in the lymphedema management program, Khurda District, Odisha State, India, July 2009–July 2011.

	Baseline (N = 370)		6 Months (N = 324)		12 Months (N = 321)		18 Months (N = 332)		24 Months (N = 316)		P-value*
	N	%	N	%	N	%	N	%	N	%	
Overall program compliance†	10	2.70	241	74.38	213	66.36	211	63.55	200	63.29	<0.0001
Knowledge on how to care for lymphedema	66	17.84	324	100.00	320	100.00	332	100.00	316	100.00	<0.0001
Difficulty accessing clean water	77	20.87	8	2.47	14	4.36	30	9.04	32	10.13	0.0001
Difficulty accessing soap	187	50.54	16	4.94	16	4.98	16	4.98	42	13.29	<0.0001
Difficulty accessing cream	185	50.00	20	6.17	18	5.61	50	15.06	41	12.97	<0.0001
Difficulty accessing oral medicine during ADLA	205	55.41	110	33.95	119	37.07	162	48.80	187	59.18	0.2849
Difficulty accessing the hospital	226	61.08	138	42.59	159	49.53	174	52.41	188	59.49	0.5477
Own mosquito net	306	82.70	241	74.38	235	73.21	258	77.71	251	79.43	0.2743
Presence of inter-digital entry lesions	100	27.03	79	24.38	92	28.66	85	25.60	87	27.53	0.8936
Number of MDAs (Mean, SD)	2.20	1.50	2.20	1.40	2.20	1.40	2.10	1.50	3.20	1.70	<0.0001
ADLA episodes in the past 30 days (Mean, SD)	0.42	0.92	0.14	0.45	0.23	0.71	0.26	0.72	0.23	0.69	0.0032
ADLA episodes in the past 6 months (Mean, SD)	1.06	1.60	0.62	1.15	0.55	1.00	0.71	1.27	0.65	1.39	0.0007
Disability assessment score (Mean, SD)	66.18	22.76	58.21	16.36	60.08	18.00	63.85	19.68	60.41	19.04	0.0004

*Denotes the p-value for the difference between baseline and 24 months.

† Program compliance was measured through a weighted compliance score to all techniques and dichotomized into two groups: compliant and non-compliant. Scores were weighted by the presence of inter-digital entry lesions.

### Predictors of Compliance

Table 5 displays the frequency of potential predictors of compliance during the course of the study at 6 month time intervals. Patients' perceived knowledge on how to care for their lymphedema reached 100% at 6 months and remained for the rest of the study period. The mean number of MDAs in which patients reported participating increased significantly over time. Difficulty accessing water, soap, and antifungal cream, decreased significantly over time as did the mean number of ADLA episodes and perceived disability assessment score.

In bivariate models, disease severity was associated with compliance to several individual lymphedema management techniques (data not shown). Those with advanced lymphedema at baseline (OR = 5.96 (95% CI: 4.16, 8.54)) and those with inter-digital entry lesions (OR = 3.49 (95% CI: 2.82, 4.30)) were more likely to comply with antifungal cream. Those with advanced lymphedema were less likely to exercise (OR = 0.63 (95% CI: 0.45, 0.88)). Patients with advanced lymphedema at baseline (OR = 0.39 (95% CI: 0.23, 0.66)) and with entry lesions (OR = 0.70 (95% CI: 0.55, 0.90)) were less likely to comply with wearing footwear outside.

Increasing age, increasing number of ADLA episodes, difficulty accessing water, soap, antifungal cream, antibiotics, and the hospital as well as increasing disability assessment score were negatively associated with overall compliance to lymphedema management techniques in bivariate models (Table 6). Having at least a primary school education was positively associated with compliance. In a multivariable logistic model (Table 6) difficulty accessing soap and antifungal cream were negatively associated with compliance to lymphedema management techniques.

**Table 6:** Predictors of compliance to lymphedema management techniques among patients enrolled in a lymphedema management program, Khurda District, Odisha State, India, July 2009–July 2011.

Predictor	Unadjusted OR (95% CI)	Adjusted OR (95% CI)
Age	0.98 (0.97, 0.99)*	0.98 (0.97, 0.989*
Male gender	1.18 (0.94, 1.47)	
≥ Primary school education	1.26 (1.01, 1.58)*	1.19 (0.90, 1.57)
Paid work status	1.07 (0.87, 1.31)	
Owning mosquito net	1.04 (0.86, 1.27)	
Presence of chronic conditions†	1.13 (0.95, 1.33)	
Number of ADLA episodes (30 day)	0.89 (0.79, 0.99)*	0.98 (0.81, 1.19)
Number of ADLA episodes (6 month)	0.90 (0.84, 0.95)*	0.92 (0.84, 1.01)
Difficult access to water**	0.47 (0.35, 0.63)*	1.09 (0.74, 1.63)
Difficult access to soap**	0.21 (0.16, 0.27)*	0.39 (0.27, 0.56)*
Difficult access to antifungal cream**	0.23 (0.18, 0.28)*	0.35 (0.26, 0.47)*
Difficult access to antibiotics**	0.72 (0.62, 0.83)*	0.93 (0.74, 1.18)
Difficult access to the hospital**	0.77 (0.66, 0.89)*	0.96 (0.76, 1.22)
Increasing disability assessment score	0.99 (0.98, 0.99)*	1.00 (1.00, 1.01)
Presence of entry lesions	1.18 (0.97, 1.43)	
Advanced lymphedema stage at baseline	1.16 (0.84, 1.59)	
Baseline ADLA rate	0.29 (0.01, 17.08)	

*P-value <0.05.

**Difficult vs. not difficult.

† Other chronic conditions include high blood pressure, diabetes, cancer, heart problems, stomach problems.

### Compliance to Lymphedema Management Techniques and Rate of ADLA Episodes

The association between overall compliance to lymphedema management techniques measured through the compliance score and the 30-day rate of ADLA episodes was assessed. Significant interaction by presence of entry lesions at the previous time point was present. Overall compliance to lymphedema management appeared to have little to no association with the frequency of ADLA episodes among those without entry lesions (RR = 0.87 (0.69, 1.10) (Table 7). Among those with entry lesions, compliance was associated with an increase in the rate of ADLA episodes (RR = 1.44 (1.11, 1.86)). To explore this association further, the compliance score was lagged by two time points (data not shown) in order to allow enough time for the potential impacts of lymphedema management to take place. Using this technique, compliance was associated with a decrease in the rate of ADLA episodes among those with entry lesions (RR = 0.77 (95% CI: 0.59, 0.99)) and was somewhat associated with a decrease in the rate among those without entry lesions (RR = 0.83 (95% CI: 0.64, 1.06)), although this finding was not significant.

**Table 7:** Association between compliance to lymphedema management techniques at the previous time point and 30-day rate of ADLA episodes, Khurda District, Odisha State, India, July 2009–July 2011.

Technique	Rate Ratio (95% CI)*
**Overall compliance** ^γ?^	
Entry Lesions‡	1.44 (1.11, 1.86)
No Entry Lesions‡	0.87 (0.69, 1.10)
**Soap** ^¥δ^	
Entry Lesions‡, Advanced Lymphedema†	0.67 (0.43, 1.04)
Entry Lesions‡, Early Lymphedema†	1.45 (0.91, 2.29)
No Entry Lesions‡, Advanced Lymphedema†	0.26 (0.15, 0.45)
No Entry Lesions‡, Early Lymphedema†	0.57 (0.42, 0.77)
**Antifungal cream** φ	
Entry Lesions‡	1.09 (0.81, 1.46)
**Footwear outside** #	1.04 (0.85, 1.26)

*Adjusted results of Poisson regression for correlated data. Each rate ratio is comparing the rate of ADLA episodes among those who are compliant to the rate among those who are not compliant within the specified clinical disease groups.

γ Dichotomized compliance score summarizing compliance to soap, cream, elevation, exercise, and footwear. Adjusted for baseline lymphedema status (advanced vs. early), access to water, access to soap, access to cream, access to the hospital, and number of time patient participated in MDA.

? Interaction with inter-digital entry lesions at previous time point was statistically significant.

‡ Inter-digital entry lesions at previous time point.

¥ Interaction with inter-digital entry lesions at previous time point and baseline lymphedema status statistically significant.

δ Adjusted for access to water, access to soap, access to cream, access to the hospital, number of time patient participated in MDA, and compliance to all other techniques.

† Lymphedema status at baseline.

φ Only among those with inter-digital entry lesions at the previous time point. Adjusted for access to water, access to soap, access to cream, access to the hospital, number of time patient participated in MDA, lymphedema status at baseline (advanced vs. early), and compliance to all other techniques.

# Adjusted for baseline lymphedema status (advanced vs. early), inter-digital entry lesions at the previous time point, access to water, access to soap, access to cream, access to the hospital, number of time patient participated in MDA, and compliance to all other techniques.

Focusing on techniques that target the prevention of ADLA episodes, the relationships between compliance to specific techniques at a previous time point and ADLA episodes were also assessed. Exploring compliance to soap, there was significant interaction by the presence of inter-digital entry lesions at the previous time point and baseline lymphedema stage (advanced vs. early). Compliance to soap was significantly associated with a decreased rate of ADLA episodes among those without inter-digital entry lesions for patients with both early and advanced baseline lymphedema (Table 7). Since antifungal cream was only indicated among those with inter-digital entry lesions, the association between compliance to cream use and ADLA episodes was evaluated only among those with inter-digital entry lesions present at the previous time point. Compliance to antifungal cream use had little to no association with the rate of ADLA (Table 7). Lagging compliance to antifungal cream two time points (data not shown), the rate ratio was further from the null, but was not significantly associated with a lower rate of ADLA episodes, RR = 0.88 (0.63, 1.22) (only among those with lesions). Compliance to wearing footwear outside had a little to no association with the rate of ADLA episode (Table 7).

### Compliance to Lymphedema Management Techniques and Lymphedema Progression

When exploring the association between overall compliance to lymphedema management techniques and lymphedema progression from one time point to the next, those who were compliant to all management techniques were less likely to progress to a more advanced stage (OR = 0.84 (0.62, 1.12)) (Table 8), yet the OR was not statistically significant. We also examined compliance to specific techniques at a previous time point that target the prevention of lymphedema progression. Compliance to soap was negatively associated with lymphedema progression (RR = 0.63 (0.41, 0.98)). Compliance to antifungal cream at the previous time point, only among those with inter-digital lesions present at the previous time point, was associated with a decrease in the odds of lymphedema progression, although the finding was not statistically significant (Table 8). Compliance to elevation and exercise of the limb and wearing footwear outside had little to no association with lymphedema progression.

**Table 8:** Association between compliance to lymphedema management techniques and lymphedema progression since previous time point. Khurda District, Odisha State, India, July 2009–July 2011.

Technique	OR (95% CI)*
**Overall compliance** φ	0.84 (0.62, 1.12)
**Soap** †	0.63 (0.41, 0.98)
**Antifungal cream** γ	
Entry Lesions‡	0.53 (0.21, 1.29)
**Elevate** †	1.11 (0.74, 1.67)
**Exercise** †	1.04 (0.74, 1.47)
**Footwear outside** †	0.93 (0.69, 1.26)

*Adjusted results of logistic regression for correlated data. Each rate ratio is comparing the rate of ADLA episodes among those who are compliant to the rate among those who are not compliant within the specified clinical disease groups.

φ Dichotomized compliance score summarizing compliance to soap, cream, elevation, exercise, and footwear. Adjusted for baseline lymphedema status (advanced vs. early), inter-digital entry lesions at the previous time point, and number of time patient participated in MDA.

† Adjusted for baseline lymphedema status (advanced vs. early), inter-digital entry lesions at the previous time point, number of time patient participated in MDA, and compliance to all other techniques.

γ Only among those with inter-digital entry lesions at the previous time point. Adjusted for baseline lymphedema status (advanced vs. early), number of time patient participated in MDA, and compliance to soap, elevation, exercise, and wearing footwear.

‡ Inter-digital entry lesions at previous time point.

## Discussion

This study found that patients enrolled in a community-based lymphedema management program experienced a 35% lower rate of ADLA episodes at 24 months compared to baseline. The rate of ADLA was lowest six months after enrollment, yet a decrease was sustained over the two year period of the study. These findings are consistent with other studies which have also found a decrease in ADLA episodes following enrollment in a lymphedema management program [2], [3], [13], [14], [23], [24]. Other programs have found a similar plateauing of the ADLA rate 3–12 months after beginning lymphedema management [13], [14]. Although our results show a significant decrease in the rate of ADLA episodes over the 2 years since the lymphedema management program was implemented, we recognize that this decrease may be partially influenced by patient receipt of anti-filarial drugs during the two MDAs that took place over the course of the study [21]. To control for this possibility, we included the number of MDAs in which each patient reported ever participating in our multivariable model exploring the effectiveness of the program over time. In doing this, we found that the rate of ADLA episodes at 24 months was 0.57 that of the rate at baseline. Even while controlling for MDA in our analyses, we do note the possibility for the decrease in ADLA episodes to be partially influenced by decreased LF transmission as seen by others [21]. Data obtained from the Odisha State Ministry of Health for the entire state illustrate a decrease in the microfilaremia from 0.69% in 2009 to 0.42% in 2011 (personal communication). These data, however, are for the entire state of Odisha, and may not represent transmission levels in the district of Khurda.

The rise in ADLA episodes after the 6 month time point may be partially explained by the fact that following 6 months in the program, patients were expected to procure their own soap and anti-fungal cream from local stores and pharmacies. Accompanying the dip in ADLA rate at 6 months and subsequent rise, reported compliance to program techniques peaked at 6 months with a subsequent decline and plateau through 24 months, providing further evidence for this possible explanation.

Patients with advanced lymphedema at baseline and inter-digital entry lesions at each time point had the highest rate of ADLA episodes throughout most of the study period. Although several studies have found an increased ADLA rate among those with increasing lymphedema stage [6], [13], [23], [25] and among those with inter-digital entry lesions [23], [25], [26], none have found an interaction between the disease groups. The decrease in ADLA rate was most striking among patients with advanced lymphedema at baseline and no entry lesions, suggesting the program was most impactful among these patients for decreasing ADLA episodes.

This study demonstrates that patients enrolled in the lymphedema management program experienced a moderate decrease in lymphedema stage. Given the concerns that advanced lymphedema may be difficult to reverse, it is not surprising that the majority of lymphedema regression occurred among patients with stages 3 and 4 transitioning to stages 1 and 2. These results are similar to the leg volume decreases found in Haiti among patients with stages 2, 3, and 4 lymphedema [13]. The percentage of patients whose lymphedema progressed to a more severe stage from one time point to the next also decreased over the study period suggesting that a basic hygiene and management program is capable of slowing the advancement of chronic lymphedema. Decreased ADLA rates and slowing the advancement of chronic lymphedema have important implications for the social stigma, debilitation, and quality of life impacts seen with LF disease. We have previously reported that patients in the lymphedema management program in Khurda district experienced a significant reduction in disability in every domain of the WHO-DAS II disability assessment score [26].

When evaluating predictors of compliance to all lymphedema management techniques, we found that reported difficulty accessing soap and antifungal cream were negatively associated with compliance indicating that access to the resources needed to perform the lymphedema management techniques may improve compliance. The study in Léogâne, Haiti found that compliance was associated with female gender and age >40 years [13], two associations we did not find. Patients enrolled in the limb-care program in Sri Lanka identified inconvenience, inability to find footwear large enough to fit, and forgetting to exercise the affected limb as the main reasons for non-compliance to the limb-care program [27].

Among patients without inter-digital entry lesions, overall compliance to lymphedema management was slightly associated with a reduced ADLA rate, but this finding was not significant. Compliance was significantly associated with an increased rate of ADLA episodes among those with entry lesions, which is difficult to explain biologically and has not been noted in other studies [13] [27]. However, when lagged two time points, compliance to lymphedema management techniques was associated with a decreased rate of ADLA episodes among both groups. This suggests that the preventative benefits of the lymphedema management techniques may not be seen until months after initiation, especially among those with entry lesions who are already at increased risk for ADLA episodes.

Looking at the lymphedema management techniques separately, compliance to washing the limb with soap decreased the rate of ADLA episodes in most disease groups. Compliance to antifungal cream among those with inter-digital entry lesions present had no effect on the rate of ADLA. This finding will need to be explored further in order to better understand the efficacy of antifungal cream use in the prevention of ADLA episodes. The only other studies evaluating antifungal cream compliance and the rate of ADLA episodes did not find an association [5], [13].

Evaluating the effects of compliance on the chronic form of LF, lymphedema progression, patients who reported overall compliance to lymphedema management had slightly lower odds of lymphedema progression. Although this finding was not statistically significant, this study was not initially powered to detect a change in lymphedema progression, rather a change in ADLA episodes. Compliance to soap had a significant negative association with the progression of lymphedema. Although studies of lymphedema management programs have found reductions in leg volume after implementation of the program [12], [13], no other studies have found an association between self-reported compliance to a lymphedema management technique and chronic lymphedema progression. One study found a slight positive association between uptake of diethylcarbamazine and ivermectin [28] and decreasing lymphedema stage. Therefore, we controlled for the frequency of MDA uptake among this population and still found a significant effect of soap compliance.

This study had several limitations. Aside from the physical examination of lymphedema stage, all results were based on patient self-report. Results involving the 30-day rate of ADLA episodes may be subject to recall bias. Patients may have inaccurately recounted the number of ADLA episodes they experienced or may have had a difficult time identifying distinct episodes. It would be ideal to have prospective information regarding ADLA episodes with detailed information on the length of the episode and if any other episodes occurred simultaneously. This would allow us to calculate a more accurate rate of ADLA episodes and to consider important aspects such as wash out period. Another major limitation of this study is the lack of a true control group: a subset of comparable patients who did not receive the community-based lymphedema program. Because of this limitation, we note the possibility that the decrease in the rate of ADLA episodes over the study period may have occurred regardless of the intervention. A control group for this study was not deemed to be feasible since it would involve withholding knowledge of lymphedema management techniques from patients with lymphedema.

In addition, this study may have been limited by a social desirability bias leading patients to overestimate compliance to the lymphedema management techniques in order to please the interviewer. Selection bias may also be present as those who agreed to participate in the study may have been more willing to work to improve their lymphedema compared to those who did not enroll in the study. When exploring the association between compliance to lymphedema management techniques and the rate of ADLA episodes, we lagged the compliance variable by one time point. This was done for both the 1 month and 6 month intervals of data points. For example, for ADLA rate at 3 months, we looked at compliance reported at the 2 month time point. For the ADLA rate at 12 months, we looked at compliance reported at the 6 month time point. We realize that the time intervals are unequal and this may be affecting the estimate of effect. Lastly, this study was conducted amongst a cohort of lymphedema patients in one district of Odisha State, India which may limit the generalizability of the results.

This study solidifies previous findings showing a decreased ADLA rate among patients enrolled in a community-based lymphedema management program and demonstrates that this decrease can be maintained over a 2-year time period. This study is one of the first to demonstrate an association between self-reported compliance to soap and the 30-day rate of ADLA episodes. We lagged the compliance variable at least one time point, in order to establish temporality of our results. In both analyses, we were able to assess the associations among different disease severity groups, explore interaction, and attempt to control for confounding. We also identified several predictors of program compliance in this population, providing information for current and future programs on how to increase compliance to lymphedema management among their patients.

Preliminary economic analyses have shown the per-person start-up cost of CASA's lymphedema management program varies from $6.5–$9.00, while the maintenance cost per person was $3.50 over the 24 months of follow-up [29]. The majority of the cost (64%) went to providing direct care to patients (i.e. training, follow-up, and supplies). Although lymphedema management programs may be more costly than annual MDA campaigns, we have demonstrated their effectiveness in this setting. In addition to the clinical benefits of the program, we have also found that persons enrolled in the program experienced a decrease of 2.5 work days lost due to their lymphedema compared to baseline [30]. These benefits were maintained after the program stopped providing supplies such as soap and anti-fungal cream at 6 months. We hope that once patients see the benefits of consistently using these supplies, they will do their best to provide for themselves. Lymphedema management programs may have somewhat higher start-up costs, but the demonstrated benefits and lower sustaining costs may outweigh them.

This study indicates that a community-based lymphedema management program is beneficial for lymphedema patients for both acute and chronic morbidity and these benefits can be sustained over a two year time period. It also illustrates that compliance to lymphedema techniques can be improved if patients are provided the proper resources such as soap and antifungal cream. Although there were differences of program effectiveness by disease severity group, we still support the broad implementation of lymphedema management efforts with integrated MDA campaigns. This work demonstrates the benefit of broad community-based lymphedema management programs implemented by volunteers or community health workers, while recognizing the need for referral care for patients with complicated lymphedema and more severe disease. Furthermore, the results suggest the clinical benefits of frequent soap use among this population. One may suggest that community-based lymphedema management programs focus strongly on the use of and continued access to soap for lymphedema patients.

This work demonstrates the benefits of scaling up lymphedema management programs, which is important since the global LF elimination program has recommended that all LF endemic countries provide access to morbidity management services [10]. Future research exploring the association between compliance and ADLA episodes as well as lymphedema progression should be performed amongst community-based lymphedema management programs in other endemic settings. Furthermore, the efficacy of antifungal cream and differences by disease severity need to be elucidated. Access to morbidity management and disability prevention services is a priority for the Global Programme to Eliminate Lymphatic Filariasis and developing cost-effective ways to provide these services will be needed even after mass drug administration for lymphatic filariasis has ended.

## Supporting Information

Checklist S1 STROBE checklist.
(DOC)
